# Literary Note

**Published:** 1899-10

**Authors:** 


### LITERARY NOTE.

THE Texas *Medical News*, one of the best medical journals in the southwest, has made great improvement of late in its appearance and make-up. It has increased its size by sixteen pages and otherwise added to its strength and influence. It is well edited, handsomely printed, and is in everyway a credit to its editors, owners and readers.

### TRANSACTIONS OF THE MEDICAL SOCIETY OF THE STATE OF NEW YORK.

A limited number of copies of the transactions of the Medical Society of the State of New York for 1899, recently issued, are available at $1.00 each, through the secretary, Dr. F. C. Curtis, Albany, the volume being sent, prepaid, on receipt of the remittance. It contains 500 pages of timely matter, many articles being by men conspicuous in the profession. This opportunity to obtain good literature at a nominal price should not be lost.

